# Rapid identification of *Aspergillus fumigatus *within the section *Fumigati*

**DOI:** 10.1186/1471-2180-11-82

**Published:** 2011-04-21

**Authors:** Rita Serrano, Leonor Gusmão, António Amorim, Ricardo Araujo

**Affiliations:** 1 IPATIMUP, Institute of Molecular Pathology and Immunology of the University of Porto, Rua Dr. Roberto Frias s/n, 4200-465 Porto, Portugal; 2 Faculty of Sciences, University of Porto, Rua do Campo Alegre s/n, 4169-007 Porto, Portugal

**Keywords:** *Aspergillus*, azole resistance, electrophoretic profile, invasive aspergillosis, molecular identification, mould, multiplex PCR, rodlet A, β-tubulin

## Abstract

**Background:**

New fungal species that are morphologically similar to *Aspergillus fumigatus *were recently described and included in section *Fumigati*. Misidentification of such fungal species, particularly of the human pathogens, *Aspergillus lentulus*, *Neosartorya fischeri*, *Neosartorya hiratsukae*, *Neosartorya pseudofischeri *and *Neosartorya udagawae*, has been increasingly reported by numerous clinical labs. Nevertheless, *A. fumigatus *still accounts for more than 90% of all invasive aspergillosis cases. The purpose of the present study was to develop a rapid method for the molecular identification of *A. fumigatus *to distinguish it from other species within the section *Fumigati*.

**Results:**

A multiplex PCR was developed using prior information based on β-tubulin (βtub) and rodlet A (rodA) partial gene sequences. PCR amplification of βtub and rodA fragments resulted in a distinctive electrophoretic pattern in *A. fumigatus *and *N. udagawae*. The polymorphisms found in the smallest amplified sequence of βtub (153 bp) and rodA (103 bp) genes were then compared among and within species of this taxonomic section. βtub was able to differentiate among 13 individual species and two groups of species that included the pathogenic fungus *A. lentulus*. A more limited number of sequences were available for rodA; nevertheless, we were able to distinguish *Aspergillus viridinutans, N. hiratsukae *and *N. udagawae*.

**Conclusions:**

The assay described in the present study proved to be specific and highly reproducible, representing a fast and economic way of targeting molecular identification of the relevant mould, *A. fumigatus*, in clinical laboratories.

## Background

Aspergillosis is the most common invasive mould disease worldwide. Recently, molecular techniques have been applied to fungal diagnosis and to the identification of species, and new fungal species that are morphologically similar to *A. fumigatus *have been described, authenticated and included in section *Fumigati *[1-3]. Therefore, this section now includes a few anamorphous *Aspergillus *species and teleomorphic species that are found in the genus *Neosartorya *[4]. The characteristics of the colonies on standard culture media are often similar to *A. fumigatus*, but conidia may be rather distinct. *Neosartorya *species produce heat-resistant ascospores [4].

Misidentification of fungal species within the section *Fumigati *has been increasingly reported by clinical laboratories. Species, such as *Aspergillus lentulus, Aspergillus viridinutans, Aspergillus fumigatiaffinis, Aspergillus fumisynnematus, Neosartorya pseudofischeri, Neosartorya hiratsukae *and *Neosartorya udagawae*, are frequently reported as *A. fumigatus *[1,2,5,6]. Some of these species have been described as human pathogens, particularly *A. lentulus*, *A. viridinutans*, *N. pseudofischeri *and *N. udagawae*, and some species have been reported to be resistant *in vitro *to the azole antifungals itraconazole, miconazole, posaconazole, ravuconazole and/or voriconazole [7,8]. Therefore, molecular identification is currently recommended for the correct identification of species within the "*A. fumigatus *complex" group. Sequencing of genes, such as actin, calmodulin, ITS, rodlet A (rodA) and/or β-tubulin (βtub), has been used to distinguish *A. fumigatus *from related species [4,9]. Multilocus sequence typing can alternatively be used for the identification of those related species, which is a strategy that also involves sequencing of several gene fragments [5]. A few other techniques, such as random amplified polymorphic DNA [10,11], restriction fragment length polymorphisms [12] and a new proposed microsphere-based Luminex assay [13], may enable molecular identification of *A. fumigatus *without sequencing. However, these methodologies are quite time consuming and labour demanding and are thus impractical in most clinical labs. In addition, they can be very expensive when employed to study collections of large numbers of isolates.

Thus, a rapid, practical and cheap alternative method for the molecular identification of *A. fumigatus *and the distinction of the species within the section *Fumigati *is required. In this study, a multiplex PCR was developed using prior information based on βtub and rodA partial gene sequences. We propose a single PCR to target the molecular recognition of the *A. fumigatus *fungus, avoiding the use of restriction enzymes. Additional sequencing of fragments of βtub and rodA allowed the identification of several *A. fumigatus *related species.

## Results

### Multiplex optimization

**Figure 1:**
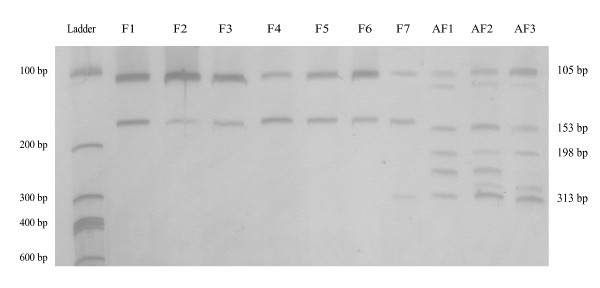
**Electrophoretic profile of several species of section *Fumigati***. F1 - *Aspergillus fumigatiaffinis*, F2 - *Aspergillus lentulus*, F3 - *Aspergillus novofumigatus*, F4 - *Aspergillus unilateralis*, F5 - *Neosartorya hiratsukae*, F6 - *Neosartorya pseudofischeri*, F7 - *Neosartorya udagawae*; AF1, AF2 and AF3 - *Aspergillus fumigatus *strains.

The present strategy was proposed to simultaneously target βtub and rodA gene fragments that are specific to a single species (*A. fumigatus*) and other gene fragments that are common to a group of species (all species of section *Fumigati*). A similar strategy was attempted with calmodulin sequences from species within the section *Fumigati*, but we could not obtain primers that were specific for *A. fumigatus *(data not shown). Thus, pairs of primers were selected based on the information on polymorphic and conserved regions of βtub and rodA genes among fungal species, as shown in Table 1 (for primer design criteria see the Methods section). As primer specificity could be improved by increasing the amplification temperature, a range from 60°C to 72°C was tested with our multiplex; highly specific primers work at high temperatures (Figure 1), whereas the amplification of some regions (e.g., the rodA region of 313 bp) could only be observed in non-*fumigatus *species at 60°C. A region of the βtub gene of 198 bp was observed only in *A. fumigatus *even when low amplification temperatures were tested. The electrophoretic profile obtained for each fungal species was very clear, revealing few secondary and/or minor bands as a consequence of primer combinations in the multiplex PCR (four nonspecific bands in the case of *A. fumigatus *and occasionally two bands in the case of non-*fumigatus *species). Those secondary bands did not reduce the performance of the multiplex PCR, as shown in Figure 1.

**Table 1:** Forward (F) and reverse (R) PCR primers employed for molecular identification of all *Aspergillus *species of section *Fumigati *and for *Aspergillus fumigatus*.

Primers (5'- 3')				Fragment length
*Aspergillus *section *Fumigati*	β-tubulin	F	AGGCAGACCATCTCTGGTGAG	153 bp
			
		R	TCGGAGGAGCCATTGTAGC	
	
	Rodlet A	F	CCAGGCTCAGCTCTCTTGCT	105 bp
			
		R	CCACCACCGATGAGGTTCTT	

*A. fumigatus*	β-tubulin	F	TGACGGGTGATTGGGATCTC	198 bp
			
		R	CGTCCGCTTCTTCCTTGTTT	
	
	Rodlet A	F	ACATTGACGAGGGCATCCTT	313 bp
			
		R	ATGAGGGAACCGCTCTGATG	

### Rapid identification of *Aspergillus fumigatus*

Multiplex PCR was successfully conducted in all fungal strains included in the study. The specificity of the primers at 69°C was confirmed by the results obtained with singleplex PCR and amplification of each gene fragment in *A. fumigatus*: partial sequences of 153 and 198 bp for βtub, and 105 and 313 bp for rodA. The electrophoretic profile with four bands (105, 153, 198 and 313 bp) was similar in all 35 tested strains of *A. fumigatus*. Non-*fumigatus *isolates of section *Fumigati*, specifically *A. fumigatiaffinis, A. lentulus*, *A. novofumigatus*, *A. unilateralis, N. hiratsukae*, *and N. pseudofischeri*, produced two discrete bands (105 and 153 bp) corresponding to the conserved region of the section *Fumigati *for which the primers were designed (as showed in Figure 1). *Neosartorya udagawae *was an exception and formed a third band (with 313 bp) in a location that was similar to the amplification of *A. fumigatus*. Amplicon sizes were confirmed using automated electrophoresis with the primers stained with 6-FAM. Therefore, the present multiplex PCR targeting βtub and rodA gene fragments resulted in a distinct band pattern in *A. fumigatus *compared to the band pattern obtained for the other species of section *Fumigati*. In addition, a clear differentiation of *N. udagawae *was also observed. The electrophoretic profile of the *Aspergillus *species of other taxonomic sections was distinct from the profile observed for *A. fumigatus *and was rarely similar to the profile obtained for species included in section *Fumigati *(two bands of 105 and 153 bp).

### Identification of species within the section *Fumigati*

**Figure 2:**
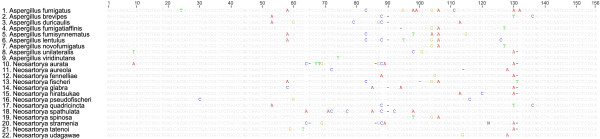
**Alignment of β-tubulin sequences from species of section *Fumigati***.

**Figure 3:**
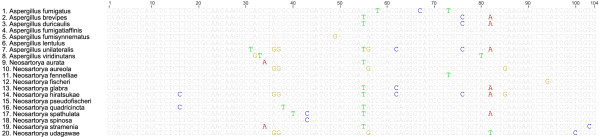
**Alignment of rodlet A sequences from species of section *Fumigati***.

The polymorphisms found in the small gene fragments of βtub (153 bp) and rodA (103 bp) were compared among and between species of section *Fumigati*. A group of 425 partial sequences of βtub and rodA from fungal species of section *Fumigati *available at GenBank and EMBL-Bank were downloaded (annotation numbers are available as supplemental data; see additional file 1). A detailed alignment of βtub and rodA sequences of the species included in section *Fumigati *is available in Figures 2 and 3. The most relevant and exclusive polymorphic sites for each species within the section *Fumigati *were registered. The 153 bp region of βtub was able to differentiate 13 fungal species of section *Fumigati *(*A. fumigatus*, *A. fumigatiaffinis*, *A. novofumigatus, N. aurata, N. aureola, N. hiratsukae*, *N. fennelliae, N. fischeri*, *N. pseudofischeri*, *N. spathulata, N. stramenia, N. tatenoi *and *N. udagawae*) and two groups of species (the first with *A. brevipes*, *A. duricaulis *and *N. quadricinta*; and the second with *A. fumisynnematus *and *A. lentulus*). The polymorphisms that were capable of distinguishing the pathogenic moulds of section *Fumigati *are detailed in Table 2. A more limited number of sequences were available for rodA (105 bp) within the section *Fumigati*; nevertheless, this small portion of DNA allowed the distinction of *A. viridinutans, N. hiratsukae *and *N. udagawae *(Table 2). Sequencing of a rodA fragment revealed no polymorphisms in *A. novofumigatus *(the information for this species was not available from the NCBI or EMBL banks).

**Table 2:** Specific nucleotide positions for identification of pathogenic species within the section *Fumigati *(inside parentheses the number of sequences studied for each species).

Species	β-tubulin sequence	Rodlet A sequence
*Aspergillus fumigatus*	T24 ^# ^(96)	Polymorphism not found (47)

*Aspergillus fumigatiaffinis*	DelG93 ^# ^(6)	Polymorphism not found (3)

Aspergillus lentulus *	T58A and C99 (48)	Polymorphism not found (39)

*Aspergillus viridinutans*	Polymorphism not found (20)	A32G or C33T (2)

*Neosartorya fennelliae*	InsA87 ^# ^or A105G ^# ^(18)	NI

*Neosartorya fischeri*	DelC99 or A131T (5)	NI

*Neosartorya hiratsukae*	G53 and G113A (10)	C55T or G62C or T76C or C82A (6)

*Neosartorya pseudofischeri*	G116C (15)	Polymorphism not found (5)

*Neosartorya udagawae*	A114G (22)	A56G or C82T (16)

* *Aspergillus fumisynnematus *may also present these β-tubulin polymorphisms but very few sequences are still available.

# Nomenclature: T24 - a thymine is present in position 24; DelG93 - deletion of the guanine in position 93; InsA87 - insertion of an adenine in position 87; A105G - replacement of an adenine by a guanine in position 105. The position numbers result from the gene alignment (Figures 2 and 3) and position 1 is located in the beginning of forward primer. (NI - not enought information, only one sequence was available).

### Recognition of low sporulating isolates

We employed the present molecular strategy to identify two low sporulating *Aspergillus *isolates that were available in our collection and are both able to grow at 45°C. The isolates showed two discrete bands of 105 and 153 bp on the electrophoretic profile with multiplex amplification. After sequencing, those isolates were identified as *A. fumigatiaffinis *(deletion of a guanine in position 93).

## Discussion

Recently, new fungal species have been identified within the section *Fumigati*, some of which have been implicated in severe cases of trabecular bone invasion and cutaneous, cerebral, liver or pulmonary aspergillosis [1,2,14-18]. These species might be primarily resistant to azole antifungals or to amphotericin B, similar to *A. fumigatiaffinis *and *A. lentulus *[7,8], whereas *A. fumigatus *is usually susceptible to the antifungals that are available for clinical treatment [19,20]. Few clinical cases of invasive aspergillosis have been reported in which the antifungal treatment was repeatedly modified until the correct identification of the fungal agent and the administration of the appropriate antifungal treatment [17,18]. Considering that *A. fumigatus *may represent a considerable part of all clinical cases of aspergillosis, molecular characterization is essential for the correct identification of species within the section *Fumigati*. In this study, we developed a multiplex PCR strategy that was able to differentiate *A. fumigatus *from all the other related species within the section *Fumigati*. We could not test all of the species of section *Fumigati*, as some of them are extremely rare. However, we believe that the present multiplex PCR can be widely used, as *A. lentulus *is more closely related to *A. fumigatus *than most species in section *Fumigati *(e.g. *A. viridinutans*) [4,5], and a distinct electrophoretic profile was observed with two strains of this species. It is expected that other species of section *Fumigati *that are genetically distant from *A. fumigatus *can be distinguished by employing this multiplex PCR (see additional file 2 in supplemental data). A simple electrophoresis profile after PCR amplification clearly separates two species, *A. fumigatus *and *N. udagawae*, from a second group of fungal isolates of section *Fumigati*. This method is furthermore amenable to automation. Compared to previously described methodologies for *A. fumigatus *identification within its section [10-13], the proposed method facilitates the molecular recognition of this species by employing a single multiplex PCR and avoiding the need for restriction enzymes and specialized equipment. This approach is cheap and simple and would be very useful in clinical labs that routinely screen and perform the molecular identification of several mould isolates. The proposed new assay proved to be specific and highly reproducible for targeting *A. fumigatus *within the section *Fumigati *and outside this section.

A list of fungal species related to *A. fumigatus *could be identified by sequencing partial regions of βtub and rodA. A group of 14 unique species and two groups of species of section *Fumigati *were distinguished by point mutations in βtub and rodA. This work presents the first record of polymorphic sites available for the rapid identification of species within the section *Fumigati *following the analysis of more than 450 βtub and rodA sequences. This list represents a practical guide for the molecular recognition of rare fungal species, and it can certainly be expanded in the near future when more sequences of βtub and rodA are available. At present, there is a limited number of sequences that are still available at GenBank and EMBL from species of section *Fumigati*, particularly for the rodA gene. The record of rodA sequences at GenBank has been improved by the addition of the information on *A. novofumigatus*.

## Conclusions

As molecular diagnosis is being increasingly employed in clinical labs [21,22] and some labs can only detect fungal DNA (culture of the fungal agent cannot be obtained), it will become increasingly important to possess molecular protocols for the identification of moulds while avoiding misidentification of fungal species. Thus, a multiplex PCR strategy is now available that can easily differentiate *A. fumigatus *and *N. udagawae *from other *fumigatus*-related species. In addition, the proposed methodology can be used in cases of low sporulating fungal isolates frequently detected in culture, as in the case of two isolates from our collection. Pathogenic species of section *Fumigati *could be identified by sequencing βtub and rodA fragments by following the list of polymorphic sites provided in this work.

Molecular identification is at present recommended for the correct identification of species within the *A. fumigatus *complex group of species. The assay described in the present study proved to be specific and highly reproducible, representing a fast and economic way of targeting molecular identification of *A. fumigatus *in clinical laboratories.

## Methods

### Fungal strains and culture conditions

A set of 35 clinical isolates of *A. fumigatus *from the Department of Microbiology, Faculty of Medicine, University of Porto, were used in this study; the reference strain, *A. fumigatus *ATCC 46645, was also included. The isolates were identified based on macroscopic and microscopic morphological characteristics, and standard mycological procedures were followed [23]. The genotype of this set of *A. fumigatus *isolates was unique, as established by a previously standardized microsatellite based multiplex PCR specially designed for this mould [24]. A second group of fungal strains of the section *Fumigati *was obtained from Centraalbureau voor Schimmelcultures (CBS): pathogenic moulds including *Aspergillus fumigatiaffinis *(CBS 117186), *Aspergillus lentulus *(CBS 116880 and CBS 117180), *Neosartorya hiratsukae *(CBS 124073), *Neosartorya pseudofischeri *(CBS 208.92), and *Neosartorya udagawae *(CBS 114217), and two non-pathogenic moulds of section Fumigati, *Aspergillus novofumigatus *(CBS 117519) and *Aspergillus unilateralis *(CBS 126.56). In addition, a third set of 12 isolates that included strains of other *Aspergillus *sections (*Aspergillus flavus, Aspergillus niger*, *Aspergillus nidulans, Aspergillus terreus *and *Aspergillus glaucus*) and two low sporulating *Aspergillus *species from our collection were included in this study. Single colonies of all fungal isolates were cultured on Sabouraud dextrose agar for 5 days at 30°C. A sodium-hydroxide-based method was used to extract DNA from fungal conidia (the protocol is available at http://www.aspergillus.org.uk/indexhome.htm?secure/laboratory_protocols). Fungal DNA was suspended in 50 μl of sterile water and frozen at -20°C.

### Molecular identification strategy

A group of 425 partial sequences of βtub and rodA from fungal species of section *Fumigati *available at GenBank and EMBL-Bank were downloaded (annotation numbers are available in Additional file 1, supplement Table A1). These sequences were aligned, and the most polymorphic and conserved regions on βtub and rodA genes were identified. In these genomic regions, two groups of PCR primers were designed: 1) general primers for the amplification of βtub and rodA gene fragments in species of section *Fumigati*, and 2) specific primers for amplification exclusively in *A. fumigatus*. The primers were selected ensuring that the resulted genomic fragments could be distinguished based on their size. The selected PCR primers are shown in Table 1.

### PCR amplification and amplicon visualization

Multiplex PCR amplification was performed in a 5 μl final volume containing 1 μL of genomic DNA (1-5 ng/μL), 2.5 μL of 2x Qiagen multiplex PCR master mix (Qiagen, Hilden, Germany) and 0.5 μL of each primer (for a 0.2 μM final concentration of each primer). After a pre-incubation at 95°C for 15 min, the amplification was performed for a total of 35 cycles as follows: denaturation at 94°C for 30 s, annealing at 69°C for 90 s, extension at 72°C for 1 min, and a final extension step of 10 min at 72°C. Singleplex PCRs were performed for the confirmation of primer specificity (a single PCR product was obtained and subsequently sequenced). Singleplex PCR amplifications were performed using the same conditions as for the multiplex amplification. Amplicons were visualized following electrophoresis in polyacrylamide gels with a standard DNA silver staining method [25].

Amplicon sizes were confirmed with automated electrophoresis. A volume of 0.5 μL of the internal size standard GeneScan 500 LIZ (Applied Biosystems, Foster City, CA, USA) and 12 μL of HiDi formamide (Applied Biosystems) were added to the PCR amplified products (6-FAM stained forward primers were used) and processed with an ABI PRISM 3100 Genetic Analyser 16-capillary electrophoresis system (Applied Biosystems).

### DNA sequencing conditions

PCR-generated fragments were purified with ExoSAP-IT (USB Corporation, Cleveland, Ohio, USA), and the reactions were conducted with an ABI Big Dye terminator cycle sequencing kit (Applied Biosystems) under the following conditions: after a 95°C pre-incubation step of 15 min and DNA denaturation at 96°C for 15 s, 35 PCR cycles were performed with primer annealing at 50°C for 9 s, an extension at 60°C for 2 min and a final extension at 60°C for 10 min. A volume of 8 μL of HiDi formamide was added to the sequencing products, which were processed in an ABI PRISM 3100 Genetic Analyser 16-capillary electrophoresis system. The results were analyzed using the Sequencing 5.2 analysis software (Applied Biosystems).

### Data analysis

βtub and rodA partial sequences available in NCBI and EMBL for *Aspergillus *species of section *Fumigati *were aligned and compared employing the Geneious software v4.7 (Biomatters Ltd, Auckland, New Zealand) and BioEdit sequence alignment editor (available at http://www.ctu.edu.vn/~dvxe/Bioinformatic/Software/BioEdit.htm). Sequencing results from this study, which included sequences from several *A. fumigatus *isolates and from ten strains of section *Fumigati*, were added to a final database that included all partial sequences of βtub and rodA genes. Based on comparisons of all of the aligned sequences, polymorphic sites that were able to discriminate different fungal species were identified.

## Authors' contributions

RS and RA carried out the experimental studies and sequence alignment. LG, AA and RA conceived the study, participated in its design and coordination and drafted the manuscript. All authors read and approved the final manuscript.

## Supplementary Material

Additional file 1 **Accession numbers of DNA sequences**. The list of all DNA sequences included in this study that were obtained from GenBank and EMBL-Bank. Click here for file

Additional file 2 **Alignment of β-tubulin and Rodlet A primers selected for amplification of *Aspergillus fumigatus *in other species of section *Fumigati***. The polymorphic positions identified in species of section *Fumigati *considering the region of the primers designed for *A. fumigatus*. Click here for file
