# Supplementary material for: Rapid identification of Aspergillus fumigatus within the section Fumigati
Source: BMC Microbiol. 2011 Apr 21;11:82. doi: 10.1186/1471-2180-11-82 (PMC3102036; doi:10.1186/1471-2180-11-82)
Supplement: Additional file 1 — Accession numbers of DNA sequences. The list of all DNA sequences included in this study that were obtained from GenBank and EMBL-Bank. [file 1471-2180-11-82-S1.PDF]

Supplementary table. Accession numbers of DNA sequences included in this study.

GenBank β-tubulin	AB248059.1; AB248060.1; AB248061.1; AB248062.1; AB248063.1; AB248064.1; AB248065.1; AB248066.1; AB248067.1; AB248068.1; AB248069.1; AB248070.1; AF057315.2; AY048754.1; AY685147.1; AY685148.1; AY685149.1; AY685150.1; AY685151.1; AY685152.1; AY685153.1; AY685154.1; AY685155.1; AY685156.1; AY685157.1; AY685158.1; AY685159.1; AY685160.1; AY685161.1; AY685162.1; AY685163.1; AY685164.1; AY685165.1; AY685166.1; AY685167.1; AY685168.1; AY685169.1; DQ438501.1; DQ438501.1; DQ438508.1; DQ438509.1; DQ438510.1; DQ438511.1; DQ438512.1; DQ438513.1; DQ438514.1; DQ438515.1; DQ438516.1; DQ438517.1; DQ438518.1; DQ438519.1; DQ438520.1; DQ438521.1; DQ438522.1; DQ438523.1; DQ438524.1; DQ438525.1; DQ438532.1; DQ438533.1; DQ438534.1; DQ438540.1; DQ438541.1; DQ438542.1; DQ438547.1; DQ438548.1; DQ438549.1; DQ438550.1; DQ438551.1; DQ438554.1; DQ438555.1; DQ438557.1; DQ438558.1; DQ438559.1; DQ534076.1; DQ534077.1; DQ534078.1; DQ534079.1; EF669791.1; EF669792.1; EF669793.1; EF669841.1; EF669845.1; EF669851.1; EF669854.1; EU254415.1; EU310845.1; EU310846.1; EU310847.1; EU310855.1; EU310856.1; FJ609209.1; FJ624459.1; GQ376140.1; GQ376141.1; GQ376142.1; AB248073.1; AB248074.1; AB248075.1; AY738513.1; AY738517.1; AY738520.1; AY738523.1; DQ011685.1; DQ011686.1; DQ058385.1; DQ058386.1; DQ058387.1; DQ058388.1; DQ058389.1; DQ058390.1; DQ438503.1; DQ438504.1; DQ438505.1; DQ438507.1; DQ438527.1; DQ438530.1; DQ438531.1; DQ438543.1; DQ438544.1; DQ438546.1; DQ438552.1; DQ438553.1; DQ438556.1; DQ438560.1; DQ438561.1; DQ534081.1; EF669824.1; EF669825.1; EF669826.1; EU254411.1; EU310839.1; EU310842.1; EU310850.1; EU310851.1; EU310852.1; EU310853.1; EU310854.1; EU310864.1; EU310865.1; EU310866.1; EU310867.1; EU310868.1; EU310869.1; EU310870.1; AB248298.1; AB248299.1; AF057317.2; AF134775.1; AF134776.1; AF134777.1; AF134778.1; AF134779.1; AF134780.1; AY590129.1; AY590130.1; AY590131.1; AY685178.1; DQ534153.1; EF661252.1; EF669834.1; EU310843.1; EU310871.1; GQ144440.1; GQ144441.1; AF057318.1; EF669835.1; EF669836.1; AF057319.1; DQ534157.1; EF669808.1; EF669810.1; EU014109.1; AF057322.1; EF669796.1; EF669828.1; EF669831.1; EF669839.1; AF057323.1; EF669798.1; EF669811.1; EF669821.1; EF669833.1; EU014107.1; AB248300.1; AF057311.1; AY685179.1; EF669812.1; AF057313.1; EF669827.1; DQ094884.1; DQ094885.1; EU310841.1; EU310844.1; GQ376143.1; AB248067.1; AB248076.1; AB248077.1; AB248078.1; EU310862.1; AF057320.1; AF057321.1; DQ534082.1; DQ534083.1; DQ534084.1; DQ534085.1; DQ534086.1; DQ534087.1; DQ534088.1; DQ534089.1; DQ534090.1; DQ534091.1; DQ534092.1; DQ534093.1;

	DQ534094.1; EF669817.1; EF669850.1; EU014108.1; AF057324.1; DQ534095.1; DQ534096.1; DQ534097.1; EU310848.1; EU310849.1; EU310857.1; EU310858.1; EU310863.1; AF057325.1; EF669790.1; EF669795.1; EF669797.1; EF669799.1; EF669801.1; EF669805.1; EF669823.1; EU310840.1; EU310859.1; EU310860.1; EU310861.1; AF057326.1; DQ534098.1; DQ534099.1; DQ534100.1; DQ534101.1; DQ534158.1; DQ534159.1; EF669806.1; EF669807.1; EF669820.1; EF669832.1; AB248302.1; AB248303.1; DQ058391.1; DQ058392.1; DQ058393.1; DQ058394.1; DQ058395.1; DQ058396.1; DQ058397.1; DQ058398.1; DQ058399.1; DQ058400.1; DQ438526.1; DQ438529.1; DQ438535.1; DQ438536.1; DQ438537.1; DQ534080.1; DQ534102.1; DQ534103.1; DQ534161.1; DQ094886.1; DQ094887.1; AB248301.1; AF057316.1; AY685180.1; EF669852.1; AF057327.1; AF057328.1; EF669803.1; EF669804.1; AF057329.1; EF669800.1; EF669813.1; EF669816.1; EF669822.1; EF669829.1; EF669844.1; AF057330.1; EF669840.1; DQ534160.1; EF669838.1
GeneBank Rodlet A	DQ439733.1; DQ439740.1; DQ439741.1; DQ439742.1; DQ439743.1; DQ439744.1; DQ439745.1; DQ439746.1; DQ439747.1; DQ439748.1; DQ439749.1; DQ439750.1; DQ439751.1; DQ439752.1; DQ439753.1; DQ439754.1; DQ439755.1; DQ439756.1; DQ439757.1; DQ439764.1; DQ439765.1; DQ439766.1; DQ439772.1; DQ439773.1; DQ439774.1; DQ439777.1; DQ439778.1; DQ439779.1; DQ439780.1; DQ439781.1; DQ439782.1; DQ439783.1; DQ439784.1; DQ439785.1; DQ439786.1; DQ439787.1; EU254416.1; EU310812.1; EU310813.1; EU310814.1; EU310822.1; EU310823.1; EU310808.1; EU310811.1; DQ439742.1; EU310829.1; EU310810.1; EU310838.1; EU310815.1; EU310816.1; EU310824.1; EU310825.1; EU310830.1; EU310807.1; EU310826.1; EU310827.1; EU310828.1; AY738514.1; AY738519.1; AY738522.1; AY738525.1; DQ058369.1; DQ058370.1; DQ058371.1; DQ058372.1; DQ058373.1; DQ058374.1; DQ439735.1; DQ439736.1; DQ439737.1; DQ439738.1; DQ439739.1; DQ439759.1; DQ439762.1; DQ439763.1; DQ439775.1; DQ439776.1; DQ439788.1; DQ439789.1; EU254412.1; EU310806.1; EU310809.1; EU310817.1; EU310818.1; EU310819.1; EU310820.1; EU310821.1; EU310831.1; EU310832.1; EU310833.1; EU310834.1; EU310835.1; EU310836.1; EU310837.1; DQ058375.1; DQ058376.1; DQ058377.1; DQ058378.1; DQ058379.1; DQ058380.1; DQ058381.1; DQ058382.1; DQ058383.1; DQ058384.1; DQ439758.1; DQ439761.1; DQ439767.2; DQ439768.1; DQ439769.1; FJ830678.1; FJ830679.1; FJ830680.1; FJ830681.1
EMBL Rodlet A	AF057331.1; AF057333.1; AF057336.1; AB250103.1; AF057338.1; AF057339.1; AF057340.1; AF057341.1; AF057342.1; AF057343.1; AF057344.1; AF057345.1; AF057346.1; AF057347.1; AF057348.1; AF057349.1; AF057350.1; AB249898.1; AB249899.1; AB250101.1; AB250102.1
