# Supplementary material for: Rapid identification of Aspergillus fumigatus within the section Fumigati
Source: BMC Microbiol. 2011 Apr 21;11:82. doi: 10.1186/1471-2180-11-82 (PMC3102036; doi:10.1186/1471-2180-11-82)
Supplement: Additional file 2 — Alignment of β-tubulin and Rodlet A primers selected for amplification of Aspergillus fumigatus in other species of section Fumigati. The polymorphic positions identified in species of section Fumigati considering the region of the primers designed for A. fumigatus. [file 1471-2180-11-82-S2.PDF]

Additional file 2. Alignment of β-tubulin and Rodlet A primers selected for amplification of *Aspergillus fumigatus* in other species of section *Fumigati.* (Primers in direction 5’-3’.)

	β-tubulin: forward primer	β-tubulin: reverse primer	Rodlet A: forward primer	Rodlet A: reverse primer
	1 10 20	1 10 20	1 10 20	1 10 20
Aspergillus fumigatus	TGACGGGTGATTGGGATCTC	AAACAAGGAAGAAGCGGACG	ACATTGACGAGGGCATCCTT	CATCAGAGCGGTTCCCTCAT
Aspergillus brevipes	TGATGGAAGATTAGGACCAG	AGGCAAGCAGGAAGAGGACG	ATATCGACGAGGGTATCCTC	CATCAGAGCGGCTCCCTCAT
Aspergillus duricaulis	TGATGGAAAATCAGGACCAG	AGGCAAGCAGGAAGAGAACG	ATATCGACGAGGGTATCCTC	CATCAGAGCGGCTCCCTCAT
Aspergillus fumigatiaffinis	TGATGGGAGATAGGAACCTG	AGGCAAGGAAGAAGAGGACG	ACATCGACGAGGGTATCCTC	CACCAGAGCGGTTCCCTCAT
Aspergillus fumisynnematus	TGATGGGAGATTGGGACCTG	ATGCAAGAAAGAAGAGGAGG	ACATCGACGAGGGTATCCTC	CAACAGAGCGGCTCCCTCAT
Aspergillus lentulus	TGATGGGAGATTGGGACCTG	AGGCAAGGAAGAAGAGGACG	ACATCGACGAGGGTATCCTC	CAACAGAGCGGCTCCCTCAT
Aspergillus unilateralis	TGATAGGACATTAGGACCGG	ACGCGAGCAGGAAGAGGACG	ATGTCGACCAGGGTATCCTC	CACCAGAGCGACTCCCTCAT
Aspergillus viridinutans	TGATGGGAGATTAGGACCTG	AGGCAAGCAGGAAGAGGACG	ACATCGACGAGGGTATCCTC	CACCAGAGCAACTCCCTCAT
Neosartorya aurata	TGATGGAAGATTAGAACCAG	AGGCAAGCAGGAAGAGGACG	ATATCGACGAGGGTATCCTC	CATCAGAGCGACTCCCTCAT
Neosartorya aureola	TGATGGGAGATTAGGACCTG	AGGCAAGCAGGAAGA-GGCG	ACGTCGACGAGGGTATCCTC	CATCAGAGCGGCTCCCTCAT
Neosartorya fennelliae	TGATGGAAGATTAGGACCAG	AGGCAAGCGGGAAGAGGACG	ACATCGACGAGGGTATCCTT	GAGCAGAGCGACTCCCTCA
Neosartorya fischeri	TGACGGGAGATTGGGACCTG	AGG-AAGGAAGAAGAGGACG	ACATCGACGAGGGTATCCTC	CACCAGAACGGTTCCCTCAT
Neosartorya glabra	TGATGGAAGATTAGGACCAG	AGGCAAGCAGGAAGAGGACG	ATATCGACCAGGGTATCCTC	CTTCAGAGCGACTCCCTCAT
Neosartorya hiratsukae	TGATGGAATATTAGGACCAG	AGGCAAGCAGGAAGAGAACG	ATATCGACCAGGGTATCCTC	CATCAGAGCGACTCCCTCGT
Neosartorya pseudofischeri	TGATGGAAGATGAGGGCTAG	AGGCAAGCAGGAAGAGGACC	ACATCGACGAGGGTATCCTC	CAGCAGAGCGACTCCCTCAT
Neosartorya quadricincta	TGATGGAAGTTTAAGACCGG	AGGCAAGCAGGAAGAGGACG	ATATCGACGAGGGTATCCTC	CAACAGAGCAACTCCCTCAT
Neosartorya spathulata	TGATGGAAGATTAGGACCAG	AAGCAAGCAGGAAGAGGACG	ATATCGACGAGGGTATCCTC	CATCAGAGCGACTCCCTCAT
Neosartorya spinosa	TGATGGGAGATTAGGACCTG	ATGCAAGGAAGAAGAGGACG	ACATCGACGAGGGTATCCTC	GATCAGAGCAACTCCCTCAT
Neosartorya stramenia	TGATGGAAGATTAGAACCGG	AGGCAAGCAGGAAGAGGACG	ATATCGACGAGGGTATCCTC	CATCAGAGCGACTCCCTCAT
Neosartorya udagawae	TGATGGGAGATTAGGACCTG	AGGCAAGCAGGAAGAGGGCG	ACGTCGACGAGGGTATCCTC	CATCAGAGCGGCTCCCTCAT
